# Efficacy of microsurgical varicocelectomy in the treatment of premature ejaculation: A protocol for systematic review and meta-analysis

**DOI:** 10.1097/MD.0000000000021308

**Published:** 2020-07-31

**Authors:** Fuhao Li, Song Zhang, Hangyu Yao, Yueyue Fan, Yifeng Shen, Guangsen Li, Degui Chang

**Affiliations:** a Department of Andrology, Hospital of Chengdu University of Traditional Chinese Medicine, Chengdu, Sichuan Province; b Shanghai University of TCM, Shanghai TCM-Integrated Hospital, Shanghai, China.

**Keywords:** microsurgical varicocelectomy, premature ejaculation, protocol, treatment

## Abstract

**Introduction:**

Premature ejaculation (PE) is the most common type of sexual disorder among men which comprises a great of problems. Varicocele is defined as the dilation of the pampiniform venous plexus draining the testicle. At present, selective serotonin reuptake inhibitors antidepressants, topical anesthetics, tramadol, phosphodiesterase type 5 inhibitors are the common alternative strategy to improve PE. However, these therapeutic measures have several shortcomings and side effects. Recently, the correlation between varicocele and PE has attracted the attention of some researchers. A few studies consider microsurgical varicocelectomy can be a new remedy for PE. But it is still absent enough a great deal of convincing evidence. The study will assess the effectiveness and safety of the microsurgical varicocelectomy treatment in PE patients.

**Methods and analysis:**

Electronic databases including English databases (PubMed, MEDLINE, EMBASE, Web of Science, Cochrane Library) and Chinese databases (China National Knowledge Infrastructure, China Biology Medicine Database, Wanfang Database, VIP Database) will be searched from their inception to December 2020 to recognize related studies. All the randomized controlled trials of microsurgical varicocelectomy for the management of PE patients will be included. The potential outcome will include intravaginal ejaculation latency time, Chinese index of sexual function for premature ejaculation-5, visual analogue score, premature ejaculation diagnostic tool, success treatment rate, serum testosterone levels. We will conduct this study strictly according to the Cochrane Handbook for systematic reviews of interventions.

**Results:**

The current study is a protocol for systematic review and meta-analysis without results, and data analysis will be carried out after the protocol. We will share our findings in the February 28, 2021.

**Conclusion:**

This systematic review will provide more evidence to assess whether microsurgical varicocelectomy is an effective intervention for patients with PE. The results will be published in a public issue journal and offer the urologists and andrologists help to make clinical decisions.

**Ethics and dissemination:**

Formal ethical approval is not required in this protocol. We will collect and analyze data based on published studies, and since there are no patients involved in this study, individual privacy will not be under concerns. The results of this review will be disseminated to peer-reviewed journals or submit to related conferences.

**Protocol registration number:**

INPLASY202060058

## 1 Introduction

Premature ejaculation (PE) is the most common type of sexual disorder among men which comprises a great of problems such as significant distress, frustration, anxiety, interpersonal difficulties, and avoidance of sexual intimacy. Numerous authors agree that PE is that patients don’t have the ability to control ejaculation sufficiently for partners to enjoy sexual intercourse.^[1]^ However, due to the lack of a precise definition of PE, the International Society for Sexual Medicine provided evidence-based definitions.^[2]^ International Society for Sexual Medicine considered that intravaginal ejaculation latency time (IELT) less than 1 minute should be diagnosed as PE, but at the same time, it should be noted that there are some PE patients whose IELT can reach 90 seconds, which should be judged by clinicians.^[2]^ Recently, the prevalence of PE has been 26.67% according to the study of Benha University.^[3]^ Several studies suggest that the etiology of PE includes a range of biological and psychological factors such as luteinizing hormone, prolactin, thyroid-stimulating hormone, diabetes, testosterone,^[4,5]^ erectile dysfunction, infertility, lower urinary tract symptoms, and varicocele (VC) also have been recognized as risk factors for PE.^[6,7]^

Due to the etiology of PE is complicated and varied, PE still absents a universally accepted consensus on the pathogenesis. Over the past few decades, various researches paid attention to selective serotonin reuptake inhibitors (SSRIs) antidepressants (dapoxetine, paroxetine, fluoxetine, citalopram, escitalopram, and sertraline), topical anesthetics (TAs), tramadol, phosphodiesterase type 5 inhibitors (PDE5is), varicocelectomy, and selective oxytocin receptor antagonist.^[6–11]^ These measures have made some progress in the treatment of PE, but there are still some deficiencies. Recently, the correlation between VC and PE has attracted the attention of some researchers.^[12]^ VC is defined as the dilation of the pampiniform venous plexus draining the testicle. The primary pathologic process for VC formation is the dilation of the internal spermatic vein with the reflux of blood down into the pampiniform plexus. Despite the extensive literature on VC, the precise mechanism by which they can potentially affect sexual dysfunction and infertility remains elusive.^[13]^ In 2000, Younes put forward that VC patients with low plasma testosterone, which may increase the risk of PE.^[14]^

At present, the effectiveness of microsurgical varicocelectomy for PE remains controversial.^[6,12,15]^ This systematic review adopts the method of evidence-based medicine to analyze and evaluate clinical randomized controlled trials (RCTs) in patients with PE, to provide evidence for further enhancing the clinical curative effect on patients with PE. The study will assess the effectiveness and safety of the microsurgical varicocelectomy treatment in PE patients.

## 2 Objectives

With this systematic review and if possible meta-analysis we urge to further evaluate the effectiveness and safety of microsurgical varicocelectomy in the treatment of PE. The results will offer clinical decisions for urologists and andrologists.

## 3 Methods

The protocol was registered on the International Platform of Registered Systematic Review and Meta-analysis Protocols (registration number: INPLASY202060058), which could be available on https://inplasy.com. The content refers to the statement of the preferred reporting items for systematic review and meta-analysis protocols checklist.^[16,17]^

### 3.1 Eligibility criteria

The inclusion and exclusion criteria are as follows.

#### 3.1.1 Types of studies

All the RCTs of microsurgical varicocelectomy for the management of PE patients will be included without publication status restriction or writing language. Letters to editors, review articles, case reports, conference abstracts, cross-sectional studies, and all observational studies will be excluded.

#### 3.1.2 Participants

Inclusion criteria:

- Patients who have regular sexual life for more than half a year with a fixed sexual partner before the operation, clinically diagnosed as premature ejaculation (≥ 18 years old).
- Patients have been diagnosed with VC by physical examination and color Doppler ultrasonography of the male reproductive system.

Exclusion criteria:

- Patients who have used antidepressants, topical anesthetics, and other drugs to treat premature ejaculation within 3 months.
- Patients with a history of scrotal and spermatic cord injuries and congenital genitourinary abnormalities.
- Patients who have been operated on for VC.
- Patients with a history of tumors and diabetes in the past year.
- Patients with any other disease that may cause VC (such as external kidney tumor, hydronephrosis, etc).

#### 3.1.3 Types of interventions and controls

Experimental interventions:

The patients in the treatment group received microsurgical varicocelectomy (no restriction on the methods of operation and course of treatment).

Control interventions:

The control group could gain a placebo, no treatment, SSRIs antidepressants, TAs, PDE5is, exercise, or guideline-recommended conventional treatment.

#### 3.1.4 Types of outcome measures

Primary outcome:

1) Improvement in sexual intercourse time, as measured by the IELT, which could assess the time from when the penis is inserted into the vagina until the beginning of ejaculation.

Secondary outcomes:

1) Chinese index of sexual function for premature ejaculation-5 scores.
2) Visual analogue score, which is used to evaluate the pain of participants.
3) Premature ejaculation diagnostic tool (PEDT), whose item ranges from 0 (no problem) to 4 (very serious problem). The higher the score, the more severe the symptoms.
4) The success treatment rate (after treatment the participants comparing to the control group) and the complications rate.
5) Serum testosterone levels

### 3.2 Search strategy

#### 3.2.1 Data sources

Electronic databases including English databases (PubMed, MEDLINE, EMBASE, Web of Science, Cochrane Library) and Chinese databases (China National Knowledge Infrastructure, China Biology Medicine Database, Wanfang Database, VIP Database) will be searched from their inception to December 2020, to recognize related studies. The search strategy that will be run in the PubMed and tailored to the other database when necessary is presented in Table 1. Besides, the reference lists of review articles will be searched for any possible titles matching the inclusion criteria.

**Table 1:**
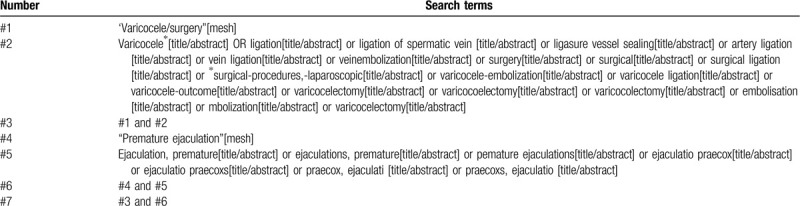
This table presents the initial draft of the search strategy with PubMed as an example. Table 1 PubMed search strategy. Table 1 example of PubMed search strategy.

#### 3.2.2 Other sources of search

The researchers will also scan the database of Chengdu University of Traditional Chinese Medicine Library and consult the experts in Urology. Dissertations of degrees will be included. The WHO International Clinical Trials Registry Platform and Google Scholar will be scrutinized for potential results. Also, the ClinicalTrials.govregistry will be explored for any unpublished trials.

### 3.3 Data extraction, quality, and validation

#### 3.3.1 Study inclusion

**Figure 1:**
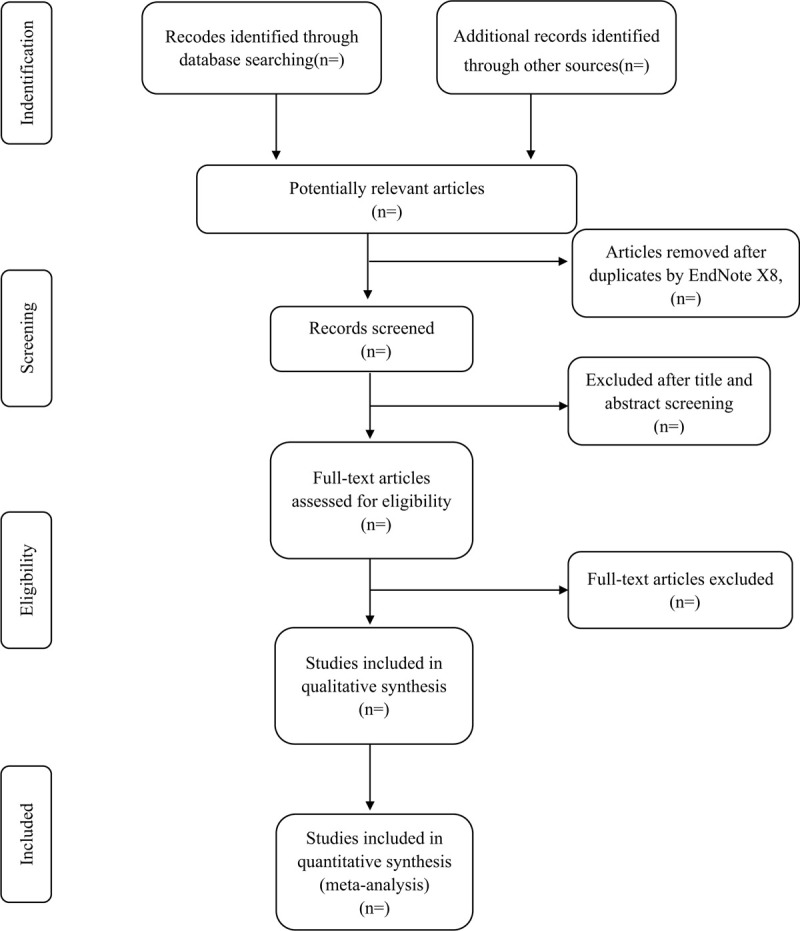
Study selection flow chart.

According to pre-defined eligibility criteria, researchers will import the literature retrieved to the Endnote X8 and eliminate the duplicate data. Studies will be removed if they do not meet the inclusion criteria. If the studies appear to meet the inclusion criteria or there is any uncertainty based on the information provided in the title and abstract, full texts will be obtained for further assessment. When necessary, we will contact the author for more details of the study to solve questions about eligibility. Two researchers will independently conduct the literature search and literature screening. Disagreements will be resolved by discussion or taking the expert (DGC) for arbitration. The number and reasons for excluding trials will be recorded in detail. A flow diagram of the study selection is shown in Figure 1.

#### 3.3.2 Data extraction and management

Upon completion of the retrieval, the 2 reviewers will independently read and extract the data from the study. Data will include the following information: title, abstract, first author and corresponding author, the country, the publication time, publications, participants, demographic characteristics (age, baby and family situation, regional, ethnic, and national), the number of participants, diagnostic criteria, types, intervention, intervention characteristics (incision length, unilateral or bilateral), observation index (IELT, Chinese index of sexual function for premature ejaculation-5, visual analogue score, premature ejaculation diagnostic tool, serum testosterone levels), the results of the study, the incidence of adverse events and type. We will use a standardized data extraction table to extract the above data. Any disagreement between the 2 reviewers will be decided by consensus or with the participation of a third reviewer. Finally, we will contact the author via email to request any missing data or clarification. If we cannot obtain the missing data, we will report it in the risk assessment of bias and consider its impact on the analysis of the data.

### 3.4 Risk of bias assessment

The risk of bias will be independently assessed by 2 reviewers and any differences will be resolved through consultation or the participation of a third reviewer. The RCTs will be evaluated using the Cochrane “risk of bias assessment” tool. The tool assesses the risk of bias mainly in the following 7 aspects: random sequence generation, allocation concealment, the blinding method for patients, researchers and outcomes assessors, incomplete result data, and selective reports. As recommended by the Cochrane manual, the risk of bias in each of these areas will be assessed as low or high depending on whether the criteria were met or not met, and the lack of information will be recorded as unclear. In most cases, disagreements will be settled by discussion between the 2 reviewers. If disagreement remained after discussion, a third reviewer will be consulted before taking the final decision on the disagreements.

### 3.5 Quantitative data synthesis and statistical methods

#### 3.5.1 Data analysis and synthesis

We will use RevMan 5.3 software (supported by Cochrane) for meta-analysis. For dichotomous data (e.g., effective and ineffective), we will calculate risk ratio (RR) and 95% confidence intervals (CIs). For continuous data, when the measurement method and unit are consistent, we will calculate the weighted mean difference and 95% CIs. When the measurement methods and units are inconsistent or the mean values of different experiments differ greatly, we will use the standardized mean difference and 95% CIs as the composite statistics.

#### 3.5.2 Investigation of heterogeneity

Heterogeneity was evaluated with χ2 test results and *I*^*2*^ statistics.^[18]^ If *P*≤.10 or *I*^*2*^≥50%, heterogeneity will be considered significant. At this point, we will use the random-effects model and conduct meta-regression or sensitivity analysis to judge the robustness of the combined results and find out the source of heterogeneity.

#### 3.5.3 Subgroup analysis

If there is significant heterogeneity in the included trials, we will identify the source of heterogeneity through subgroup analysis and manage the heterogeneity:

1) The duration and severity of VC.
2) Intervention features: unilateral varicose vein surgery or bilateral varicose vein surgery.
3) The duration and severity of PE.
4) Whether with other sexual dysfunctions.
5) Demographic characteristics of the patients: age, marital and family status, region, race, and ethnicity.
6) Follow-up time.

#### 3.5.4 Sensitivity analysis

A sensitivity analysis will be performed to test the robustness of the review result and to detect the source of heterogeneity. This can be done by excluding trials with a high risk of bias or eliminating each study individually. And, the impact of methodological quality, sample size, and missing data will be assessed. Then the analysis will be repeated after the exclusion of low methodological quality studies and the results compared with the previous meta-analysis.

#### 3.5.5 Grading the quality of evidence

Grading of recommendations assessment, development and evaluation method^[19]^ will be performed to evaluate the level of confidence in regards to outcomes. It is based on 5 key domains: risk of bias, consistency, directness, precision, and publication bias. Two independent reviewers will assess these studies. In most cases, disagreements were resolved by discussion between the 2 reviewers. If disagreement remained after discussion, the third reviewer will be consulted before taking the final decision on the disagreements.

#### 3.5.6 Publication bias

Published bias will be measured by the funnel plot. If the result is indistinct, the Begg test and Egger test will be used (by STATA software 11.0).

#### 3.5.7 Reporting of the review

The methodological quality of the systematic review and meta-analysis will be standardized by each item of the AMSTAR-2 tool.^[20]^ And the results will be reported following the preferred reporting items for systematic reviews and meta-analysis statement.^[21]^

## 4 Discussion

PE is the multifactorial disorder which not only seriously affects the physical and mental health of men, but also greatly harms the feelings between patients and partners, and exerts great burden on public health. Many different kinds of drugs were tried to improve the quality of sexual life among patients with PE, including SSRIs, TAs, tramadol, and PDE5is. But such studies suggested that these drugs with some adverse effects on the patients.^[5,7]^

In the past 20 years, some researches suggested that there is a correlation between VC and PE, which provides us with a new treatment for PE. At present, several studies have shown that microsurgical varicocelectomy can effectively improve PE, but its efficacy has not been evaluated scientifically and systematically.

The review aims to evaluate the efficacy and safety of the microsurgical varicocelectomy in patients with PE. Through this study, more detailed observation and analysis of microsurgical varicocelectomy for PE can guide surgeons to select operations more rationally and more specifically. There are some limitations to this review. As we are not good at other languages, the literature we searched is limited to Chinese and English, which will cause certain biases. Besides, the limitation of sample size also leads to the instability of conclusion reliability.

## Author contributions

GSL and DGC are the guarantors. All authors read, provided feedback, and approved the final manuscript.

**Conceptualization:** Fuhao Li, Song Zhang

**Data curation:** Hangyu Yao, Yueyue Fan

**Formal analysis**: Yifeng Shen, Hangyu Yao

**Methodology:** Fuhao Li, Song Zhang

**Project administration:** Fuhao Li, Song Zhang, Degui Chang

**Software:** Yifeng Shen, Yueyue Fan

**Supervision:** Guangsen Li, Degui Chang

**Validation:** Fuhao Li, Hangyu Yao

**Writing – original draft:** Fuhao Li, Song Zhang

**Writing – review & editing:** Guangsen Li, Degui Chang
